# Effectiveness of targeted viral load strategy in a public health HIV/AIDS programme

**DOI:** 10.1186/1471-2334-14-S3-O22

**Published:** 2014-05-27

**Authors:** Rajyasree De, Nivedita Dutta, Dolanchampa Modak, Shantasil Pain, Sripati Dasmohapatra, Sukamal Bisoi, Subhasish Kamal Guha

**Affiliations:** 1 Centre of Excellence in HIV Care, Calcutta School of Tropical Medicine, Kolkata, West Bengal, India; 2 Department of Medicine, North Bengal Medical College, Darjeeling, West Bengal, India; 3 Care, Support & Treatment Division, West Bengal State AIDS Prevention & Control Society, Kolkata, West Bengal, India; 4 Department of Community Medicine, RG Kar Medical College, Kolkata, West Bengal, India

## Background

Targeted viral load (VL) strategy is followed in the ART programme of India. Patients failing antiretroviral therapy, undergo VL testing and switched to 2ndline ART if VL is >5000 copies/ml.

## Methods

Data of patients referred to School of Tropical Medicine, Kolkata for suspected treatment failure (TF) was analyzed for the period of December2008 to November2013 to ascertain the positive predictive value (PPV) of immunologic and/or clinical parameter for confirming virologic failure (VF) and to observe the short term outcome of secondline treatment. Paired t- test was used for data analysis.

## Results

VF was noted in 287(51.3%) of 560 patients (median age 37 years; male–436(77.85%); median CD4 109 cells/mm^3^ at suspected TF. Number of patients failing by immunologic, clinical and clinicoimmunologic criteria was 414(74%), 11(2%) & 135(24%) respectively with corresponding PPV of 48%, 46% & 63% respectively. PPV of CD4 falling below baseline, >50% drop from on-treatment peak value and failure to reach 100 cells/mm^3^ after 12 months of ART was 37%, 55% and 25% respectively. Among 216 patients with minimum 6 months follow-up, the median CD4 & VL changed significantly following 6 months of secondline ART (103.5 to 232 cells/mm^3^; *p*<0.0001 and 4.04 to 2.6 log_10_ copies; *p*<0.0001 respectively). Undetectable VL was achieved in 164(76%). Out of 284 patients starting secondline ART, 25 (8.8%) died within 6 months.

## Conclusion

Immunoclinical criteria have low PPV in diagnosing VF. Despite late switch, majority (76%) could achieve undetectable VL at 6 months but the high early mortality (8.8%) is a concern.

